# Concerns about falling in people with Mild Cognitive Impairment and dementia: a scoping review of exercise interventions

**DOI:** 10.3389/frdem.2024.1456125

**Published:** 2024-11-20

**Authors:** Erica Dove, Patricia Hewston, Rosalie H. Wang, Kara K. Patterson, Arlene J. Astell

**Affiliations:** ^1^Rehabilitation Sciences Institute, Temerty Faculty of Medicine, University of Toronto, Toronto, ON, Canada; ^2^Toronto Rehabilitation Institute, KITE Research Institute, University Health Network, Toronto, ON, Canada; ^3^Department of Medicine, Geras Centre for Aging Research, McMaster University, Hamilton, ON, Canada; ^4^Department of Occupational Science and Occupational Therapy, Temerty Faculty of Medicine, University of Toronto, Toronto, ON, Canada; ^5^Department of Physical Therapy, Temerty Faculty of Medicine, University of Toronto, Toronto, ON, Canada; ^6^Psychology Department, Northumbria University, Newcastle upon Tyne, United Kingdom

**Keywords:** cognitive impairment, older adults, fall prevention, balance confidence, exercise

## Abstract

**Background:**

Concerns about falling (e.g., low balance confidence) increase fall risk in older populations with balance impairments. Exercise can improve physical limitations associated with falls (e.g., poor balance), which are more prevalent in people with Mild Cognitive Impairment (MCI) and dementia. This scoping review aimed to understand exercise interventions targeting concerns about falling in people with MCI and dementia.

**Methods:**

Using Arksey and O'Malley's five-stage scoping review framework, 968 search combinations were run across six electronic databases from inception to September 15, 2023. Articles were available in English full text, featured original peer-reviewed research with an intervention study design, targeted people with MCI or dementia with the exercise intervention, and included concerns about falling as an outcome measure.

**Results:**

Of the 2,111 articles screened, 27 met the inclusion criteria. Only one article looked at concerns about falling as a primary outcome; in the remaining studies, concerns about falling were a secondary outcome. Multi-modal interventions (i.e., containing more than one type of exercise) were most common, with balance and strength as the most frequently employed exercise types. Secondary results are presented on (i) intervention details, (ii) outcomes and measures for concerns about falling, (iii) participant accommodations, and (iv) components of effective interventions for concerns about falling.

**Conclusions:**

There is a lack of focus on concerns about falling experienced by people with MCI and dementia. Although concerns about falling were not the primary outcome of most papers, the results highlight the potential of exercise interventions to help address concerns about falling and other fall risk factors (e.g., balance, cognition) in people with MCI and dementia.

## 1 Introduction

Falls are a significant public health problem and the second leading cause of unintentional injury deaths worldwide (World Health Organization, 2021). Globally, an estimated 684,000 people die of falls each year, with the most significant proportion of fallers being older adults aged 60 years and above (World Health Organization, 2021). For people living with mild cognitive impairment (MCI) and dementia, most of whom are older adults (Sachdev et al., 2014), falls are two to eight times more likely to occur than in older adults without dementia (Meuleners et al., 2016), making cognitive impairment an independent risk factor for falling (Hsu et al., 2012).

In addition to cognition, the risk of falls in MCI and dementia is also linked to physical factors, such as balance impairments (Mazoteras Muñoz et al., 2010). For example, a study of people with subjective cognitive impairment, MCI, and Alzheimer's disease found balance impairments in all groups, with the impairment levels increasing with the severity of cognitive impairment (Tangen et al., 2014). Concerns about falling (e.g., fear of falling) are defined as “a persistent feeling related to one's risk of falling during daily activities” (Neuroscience Research Australia, 2020). These concerns are widely known to increase the occurrence of falls in older adults without cognitive impairment (Asai et al., 2022). Among older adults without cognitive impairment, concerns about falling have been associated with factors such as activity avoidance, poor perceived health, and history of multiple falls (Zijlstra et al., 2007). Fear of falling (a specific concern about falling) has also been associated with lower muscle strength and worse balance performance among community-residing older adults (Deshpande et al., 2009). Further to this, concerns about falling have been associated with cognitive impairment (Uemura et al., 2014) and can also increase fall risk in people with MCI and dementia (Borges et al., 2014).

Exercise interventions for people with MCI and dementia have primarily focused on physical outcomes, particularly considering the challenges individuals with MCI and dementia experience with recall and abstract thinking required during traditional concerns about falling assessments (Delbaere et al., 2013). To mitigate this challenge, the Iconographical Falls Efficacy Scale was developed using pictorial cues to assess individuals' concerns about falling during daily activities (Delbaere et al., 2011). This visual augmentation can be helpful for individuals with cognitive impairment, as it provides a more concrete and accessible way to express their confidence in physical activities related to falls (Delbaere et al., 2013).

A systematic review and meta-analysis of 43 clinical trials demonstrated that 2–3 h per week of supervised, multi-modal physical exercise (i.e., using more than one exercise type) improved strength, balance, mobility, and endurance in people with MCI and dementia (Lam F. M. et al., 2018). However, evidence regarding the impact of improving physical outcomes on fall prevention and risk management in people with MCI and dementia is less consistent (Burton et al., 2015; Li et al., 2021; Booth et al., 2015). Exercise could be a way to address concerns about falling in people with MCI and dementia. However, our understanding of how physical exercise interventions for fall risk may influence concerns about falling in people with MCI or dementia is limited.

This scoping review aimed to understand exercise interventions targeting concerns about falling in people with MCI and dementia. The secondary aims of this review were to explore how interventions were delivered (e.g., in-person, group-based, clinic-based), the constructs and assessment tools used to measure concerns about falling, any accommodations made for participants with MCI and dementia, and the components of effective interventions addressing concerns about falling.

## 2 Methods

### 2.1 Design

We utilized a scoping review methodology to map the current landscape of exercise interventions to target concerns about falling in people living with MCI and dementia. A five-stage scoping review framework (Arksey and O'Malley, 2005) involved (i) identifying the research question(s), (ii) study selection, (iii) screening and selecting relevant publications, (iv) charting the data, and (v) collating, summarizing, and reporting the results. In addition to (Arksey and O'Malley, 2005), we also followed the Preferred Reporting Items for Systematic Reviews and Meta-Analyses extension for scoping reviews (PRISMA-ScR) checklist (Tricco et al., 2018) to ensure rigor in review conduct and reporting.

#### 2.1.1 Identifying the research questions

The primary research question for this scoping review was: “*Are there any exercise interventions targeting concerns about falling in people with MCI and dementia?*”

Secondary review questions included the following:

1. “*How were the interventions delivered (e.g., in-person) and in what setting and format?”*
2. “*What constructs and assessment tools were used to examine concerns about falling before and after the interventions?”*
3. “*What accommodations were made to support people with MCI and dementia to participate in the interventions, if any?”*
4. “*What were components of effective interventions for addressing concerns about falling?”*

#### 2.1.2 Study selection

To identify relevant studies, 968 search combinations were systematically applied across six electronic databases accessible through an institutional library. The search strategy was developed using a modified version of the Population, Intervention, Comparator, Outcome, and Study design (PICOS) model (da Costa Santos et al., 2007) (see Table 1). The searched databases included MEDLINE (Ovid), Embase (Ovid), CINAHL (EBSCO), Ageline (EBSCO), AMED (Ovid), and PsycINFO (Ovid).

**Table 1:** Final search strategy.

**Population terms**	**Intervention terms**	**Outcome terms**
Dement^*^, mild cognitive impairment, MCI, Alzheimer^*^, cognitive decline, cognitive disorder, major neurocognitive disorder, mild neurocognitive disorder, neurocognitive disorder, memory loss, cognitive impairment	Exercise^*^, exercise therap^*^, exercise intervention, exercise program, exercise training, physical activit^*^, physical training, physical therap^*^, rehabilitation, training, intervention	Fear adj2 falling, falls-related anxiety, falls efficacy, falls self-efficacy, balance confidence, balance efficacy, balance self-efficacy, movement confidence

Each term listed under its respective column was combined within and across. Where possible, search terms were expanded to include as many synonyms as possible to ensure all word variations were captured. The search was undertaken on September 15, 2023. Hand searches of retrieved articles (e.g., checking reference lists of relevant articles) were completed to generate additional results, where possible.

Once the final search strategy was confirmed with an institutional librarian, the formal searches were run across the databases. Search results from the databases were de-duplicated to remove matching publications before the title and abstract screening. All retrieved articles were tracked through database searching and hand-searching, and the number of duplicates removed was tracked using review management software.

#### 2.1.3 Screening and selecting relevant publications

Next, each article was screened against the review's inclusion and exclusion criteria, based on an adapted version of the PICOS model (da Costa Santos et al., 2007) (see Table 2). Articles were published within any timeframe. Only studies written in English were chosen as the authors can only read and understand English. The purpose of a scoping review is to map the breadth of literature on a given topic (Arksey and O'Malley, 2005). Therefore, feasibility studies were included if they met the criteria of an exercise intervention with concerns about falling as a primary or secondary outcome.

**Table 2:** Inclusion and exclusion criteria.

	**Inclusion criteria**	**Exclusion criteria**
P: Population	•Population targeted by the exercise intervention(s) are people with MCI or dementia (any type or severity)	•Targeted population was not people with MCI or dementia
I: Intervention	•Study intervention features an exercise component	•Study does not evaluate an exercise intervention
C: Comparator	•N/A	•N/A
O: Outcomes	•Concerns about falling were included as an outcome measure	•Concerns about falling were not included as an outcome measure
S: Study design	•Article describes an empirical investigation (i.e., original research) •Article evaluates an experimental study design (e.g., RCT, quasi-experimental studies)	•Gray literature, conference abstracts, position papers, protocol papers, or review papers •Study design was not experimental (i.e., observational and correlational studies were excluded)

First, two reviewers independently screened all database hits at the title and abstract levels. Both reviewers had similar experiences and had previously published scoping reviews. Reasons for excluding articles at the title and abstract stages were determined by comparing the articles against the inclusion and exclusion criteria. Discrepancies identified were resolved through discussion between the two reviewers, with the option of bringing in a third reviewer if consensus was not achieved. If a reviewer was unsure whether an article met the eligibility criteria based on the title and abstract, the article was included in the full-text screening process to ensure that potentially relevant literature was not overlooked.

**Figure 1:**
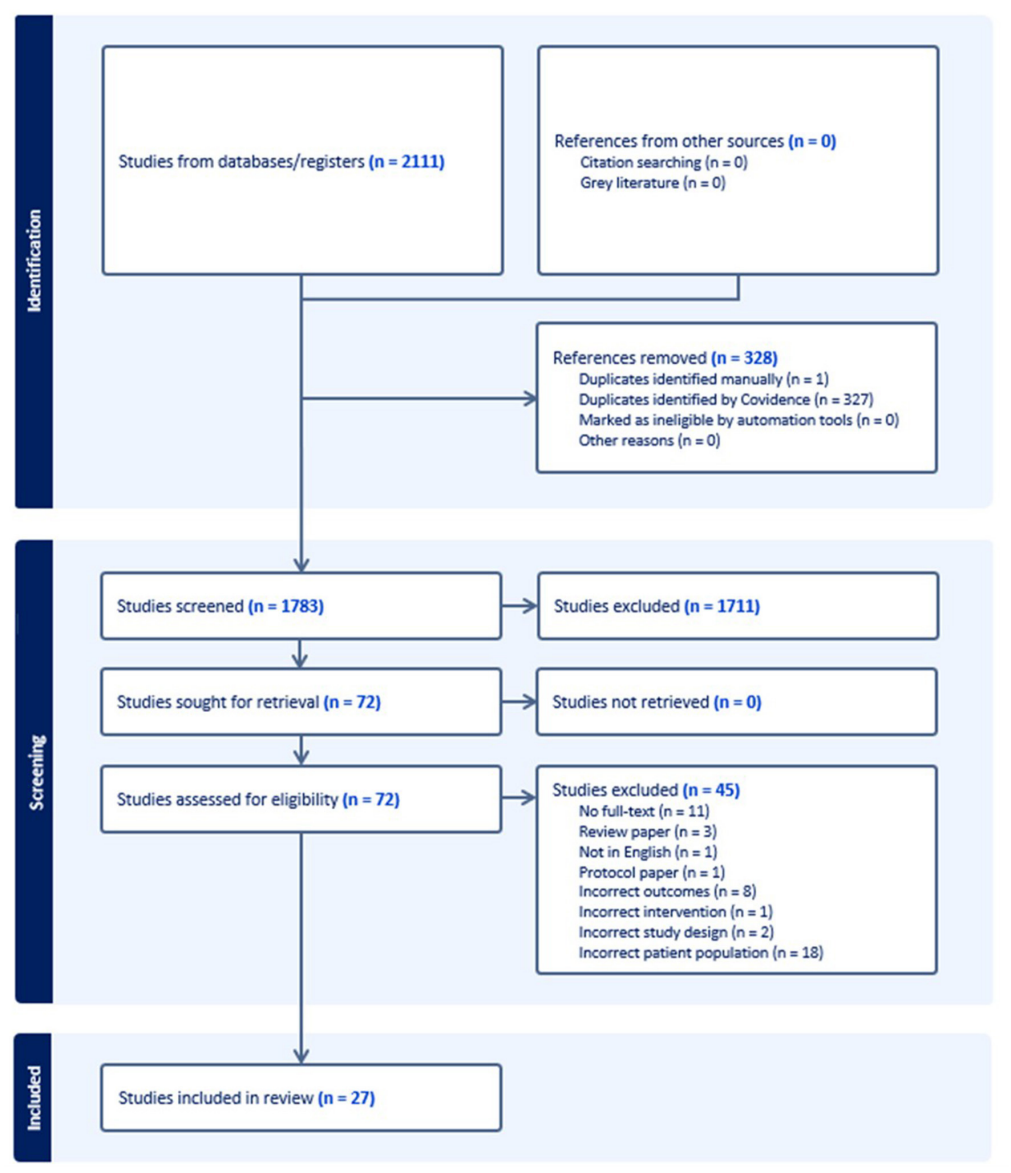
PRISMA flow diagram.

Next, the two reviewers independently reviewed full-text articles which passed title and abstract screening and collaborated to discuss their decision regarding each article and the reason behind their decision. If the reviewers disagreed, a discussion was held until consensus was achieved. Reasons for excluding articles at the full-text level were tracked using review management software. The final breakdown of studies reviewed, excluded, and included in the scoping review is presented in Figure 1 using the PRISMA diagram (Tricco et al., 2018; Peters et al., 2020).

#### 2.1.4 Charting the data

Data from the 27 included studies after full-text exclusion were charted according to publication details, study design, demographics, population targeted (i.e., MCI and/or dementia), exercise intervention employed, specific exercise types used, intervention dose, outcome measure(s) used, primary outcomes, efficacy of the interventions, delivery details (e.g., therapist-led, home-based), and accommodations for participants with cognitive impairment (e.g., cueing, practice). Data extracted from each article were synthesized to answer the research questions.

Data from the included articles were extracted by the first author using a spreadsheet and verified by the second author. Given that this review was scoping rather than systematic, a quality appraisal and risk of bias assessment of the included literature were not undertaken. Scoping reviews are meant to systematically map the breadth of the literature on a given topic rather than assess the existing literature's quality (Arksey and O'Malley, 2005; Joanna Briggs Institute, 2021; Levac et al., 2010). As such, articles were also not graded according to the strength of the evidence.

#### 2.1.5 Collating, summarizing, and reporting the results

The full-text screening results were collated, summarized, and reported by extracting data from the studies and organizing details into a table (see Table 3). Results from the 27 studies were categorized as follows: (i) exercise types, (ii) intervention details, (iii) outcome measures used to examine concerns about falling, (iv) participant accommodations, and (v) components of effective interventions.

**Table 3:** Summarized scoping review results.

**Citation**	**Location**	**Design**	**Participant descriptives (*n*, mean age [range], M/F)**	**Population (mean + SD baseline cognitive score)**	**Exercise intervention (dose)**	**Exercise Setting and Delivery (Setting, Delivery, Format, Instructor, Accommodations)**	**Types of exercises**	**Comparator**	**Outcomes**	**Concern about falling construct**	**Concern about falling outcome measure (/score)**	**Exercise pre- and post-test scores (mean)**	**Within- and between-group analyses on concerns about falling (*p* ≤ 0.05)**
Callisaya et al., 2021	Australia	RCT	*n* = 93 (44 exercise, 49 control); 72.8 years [range NR]; 39M/54F	MCI (*mean* MoCA: 26.2/30; SD: NR)	**Tablet-based exercise program** (2 h per week, 10–30 min per session, for 6 months = 52 h)	**Home-based** (Individual; remote; exercise physiologists or PTs; initial set up and tailoring, progressive increase in difficulty, monthly check-in calls)	Strength, balance, and cognitive exercises	Resources and monthly check-in calls	Primary • Gait speed Secondary • Dual-task gait speed; balance; cognition; mood; balance confidence	Balance confidence	ABC scale (/100)	• **Pre-:** 87.9 • **Post-:** 89.4	Within • NS (*p*-value NR) Between • NS (*p*-value NR)
Charras et al., 2020	France	Cross-over study	*n* = 23; 83.5 years [range NR]; 11M/12F	Dementia (*mean* MMSE: 19.21/30; SD: 6.11)	**Dance intervention** (once per week, 50 min per session, for 6 months = 21.67 h)	**ADP-based** (Group; in-person; dance teacher with nursing background led the intervention; simple sequences by imitation, caregiver involvement)	Balance and coordination exercises	No intervention	Primary • Gait; balance Secondary • Balance confidence; QoL; wellbeing	Balance confidence	ABC scale (/100)	• **Pre-:** 39.14 • **Post-:** 38.23	Within NS (*p* = 0.19) Between • Significant improvements in balance confidence (*p* = 0.02)
Chen and Pei, 2018	Taiwan	RCT	*n* = 28 (15 exercise, 13 control); 77.3 years [range NR]; 14M/14F	Dementia (*mean* MMSE: 17.15/30; SD: 5.5)	**Musical dual-task training** (once per week, 60 min per session, for 2 months = 8 h)	**Outpatient clinic-based** (Group; in-person; trained music therapist conducted the intervention)	Music-based cognitive and balance exercises	Non-music cognitive and balance activities	Primary Attentional control Secondary • Dual-task performance; balance; fear of falling; agitation	Fear of falling	FES-I (/64)	• **Pre-:** 33.4 • **Post-:** 33.1	Within • NR Between • Significant reductions in concerns about falling (*p* = 0.02)
Dechamps et al., 2009	France	RCT	*n* = 52 (26 exercise, 26 control); 80.7 years [range NR]; 22M/30F	Dementia (*mean* MMSE: 20.03/30; SD: 6.6)	**Tai Chi** (four times per week, 30 min per session, for 6 months = 52 h)	**LTC-based** (Group; in-person; trained instructor led the sessions)	Balance, strength, and flexibility exercises	Cognitive exercise program	Health-related QoL; falls efficacy; physical functioning; intervention adherence	Fall efficacy	FES (/100)	• **Pre-:** 55.3 • **Post-:** 55.1	Within • Significant improvements in fall efficacy (*p* < 0.001) Between • NS (*p* = 0.1)
Hagovska and Olekszyova, 2016	Slovak Republic	RCT	*n* = 80 (40 exercise, 40 control); 67.1 years [range NR]; 41M/39F	MCI (*mean* MMSE: 26.0/30; SD: 2.02)	**Balance and digital cognitive training** (daily, 30 min per session, for 2.5 months = 35 h)	**Outpatient clinic-based** (Format NR; in-person; intervention sessions were led by a PT; progressively increase in difficulty, individual tailoring)	Balance exercises and digital cognitive training	Balance exercises (no digital cognitive training)	Primary • Cognition Secondary • Fear of falling; balance; mobility	Fear of falling	FES-I (/64)	• **Pre-:** 18.67 • **Post-:** 11.65	Within • Significant reductions in fear of falling (*p* = 0.004) Between • Significant reductions in fear of falling (*p* = 0.001)
Harwood et al., 2023	United Kingdom	RCT	*n* = 365 (183 exercise, 182 control); 80 years [range: 65–95] 210M/155 F	Dementia and MCI (*mean* MoCA: 20.05/30; SD: 3.35)	**Dementia-specific rehabilitation program** (3 h per week for 12 months = 156 h)	**Home- and community-based** (Individual; in-person, and remote due to COVID-19; intervention led by trained clinicians; intervention tailored to individual abilities, comorbidities, interests, and goals)	Strength, balance, dual-task training, and ADL performance	Falls assessment + advice on fall prevention care	Primary • Carer-rated disability in activities of daily living for dementia Secondary • ADLs; falls; rate of falling; injurious falls; cognition; QoL; activity; frailty; functional mobility; hand grip strength; fear of falling; mood; carer burden	Fear of falling	Short FES-I (/28)	• **Pre-:** 10.4 • **Post-:** 11.0	Within • NS (*p* = 0.64) Between • NS (*p*-value NR)
Hwang et al., 2023	Taiwan	Three-arm RCT	*n* = 189 (63 CCT; 63 Tai Chi; 63 control); ≥65 years (mean and range of age NR)	MCI (*mean* MDRS: 131.2/144; SD: 12.4)	• **CCT** (three times per week; 50 min per session; for 6 months = 65 h) • **2. Tai Chi** (three times per week; 50 min per session; for 6 months = 65 h)	**Home-based** • **CCT** (Individual; in-person; intervention led by a research nurse; individualized instructions) • **2. Tai Chi** (Individual; in-person; intervention led by a Tai Chi instructor; individualized instructions)	• **CCT:** Digital cognitive training/exercise • **Tai Chi:** Balance, strength, and flexibility exercises	Health education **(HE)**	Primary • Cognition Secondary • Mobility; balance; ADL performance; balance confidence	Balance confidence	ABC (/100)	**CCT** • **Pre-:** 71.2 • **Post-:** 87.5 Tai Chi • **Pre-:** 75.1 • **Post-:** 92.3	Within • **CCT:** Significant improvements in balance confidence (*p* < 0.05) • **Tai Chi:** Significant improvements in balance confidence (*p* < 0.05) Between • Significant improvements in balance confidence in favor of Tai Chi (*p* < 0.05)
Kato et al., 2006	Japan	Quasi-experimental pre- and post-test study (two groups)	*n* = 30 (16 exercise, 14 control); 84.6 years [range NR]; 6M/24F	Dementia (*mean* HDS-R: 17.85/30; SD: 6.55)	**Fall prevention exercise program** (three times per week, 10–15 min per session, for 3 months = 9 h)	**LTC-based** (Group; in-person; intervention sessions led by trained instructors)	Flexibility, strength, and balance exercises	No intervention	Mobility; leg strength; postural sway; falls efficacy; falls	Falls efficacy	Author-modified FES (/27)	• **Pre-:** 23.6 • **Post-:** 22.1	Within • NS (*p* = 0.349) Between • NR
Kim et al., 2017	Korea	RCT	*n* = 30 (15 exercise, 15 control); 81.5 years [range NR]; 24M/6F	Dementia (*mean* MMSE-K: 15.55/30; SD: 2.65)	**Occupational therapy-centered activity program** (five times per week, 30 min per session, for 3 months = 30 h)	**ADP-based** (Group; in-person; intervention sessions led by OTs)	Balance exercises	No intervention	Cognition; fear of falling; balance; agility; leg strength; QoL	Fear of falling	FES-I (/64)	• **Pre-:** 27.5 • **Post-:** 34.9	Within • Significant reductions in concerns about fear of falling (*p* < 0.005) Between • NS (*p*-value NR)
Kiyoshi-Teo et al., 2023	United States of America	Quasi-experimental pre- and post-test study	*n* = 25; 88.6 years [range NR]; 4M/21F	Dementia and MCI (*mean* MoCA: 21.0/30; SD: 4.0)	**Multi-component, student-led fall prevention and management program** (once per week, 60 min per session, for 1.5 months = 6 h)	**Assisted living facility-based** (Individual; in-person; sessions run by supervised health professional students; individual tailoring)	Specific exercises NR (“exercise/physical therapy”)	–	Fall risk; fear of falling	Fear of falling	Short FES-I (/28)	• **Pre-:** 16.05 • **Post-:** 15.12	Within • Significant improvements in fear of falling (*p* = 0.022) Between • NA
Lam F. M. H. et al., 2018	Hong Kong	RCT	*n* = 54 (27 exercise, 27 control); 79.8 years [range NR]; 14M/40F	Dementia (*mean* C-MMSE: 14.6/30; SD: 4.6)	**Whole-body vibration and a routine exercise program** (twice per week, 30–60 min per session, for 2.25 months = 9–18 h)	**ADP-based** (Group; in-person; exercises delivered by a PT; simplified instructions)	Strength and balance exercises	Routine exercise program (without whole-body vibration)	Primary • Functional mobility Secondary • Balance; balance confidence; leg strength; QoL	Balance confidence	ABC scale (/100)	• **Pre-:** 78.1 • **Post-:** 80.0	Within • NR Between • NS (*p* = 0.757)
Lee et al., 2023	Australia	Single-group pilot feasibility study	*n* = 26; 84.5 years [range NR]; 11M/15F	Dementia (*mean* RUDAS: 16.9/30; SD: 4.7)	**Safe functional home exercise program** (three times per week, 30 min per session, for 3 months = 18 h)	**Home-based** (Individual; in-person and remote; intervention delivered by trained care staff; individualized and progressively tailored; family caregiver involvement)	Strength, balance, and dual-task exercises	–	Safety and adherence; feasibility; potential benefits (e.g., fear of falling)	Fear of falling	iconFES (/120)	• **Pre-:** 31.0 • **Post-:** 26.07	Within • Significant reductions in fear of falling (*p* < 0.01) Between • NA
Li et al., 2014	United States of America	Non-randomized pre- and post-test study	*n* = 46 (22 EG, 24 CG); 76 years [range NR]; 14M/32F	Dementia (*mean* MMSE: 23.13/30; SD: 1.18)	**Tai Chi** (two times per week, 60 min per session, for 3.5 months = 28 h)	**Community center-based** (Group; in-person; trained instructors led the intervention; practice sessions with multiple repetitions)	Balance, strength, and flexibility exercises	No intervention	Primary • Cognition Secondary • Physical performance; balance confidence	Balance confidence	Author-modified ABC scale (/5)	• **Pre-:** 3.43 • **Post-:** 4.13	Within • Significant improvements in balance confidence (*p* < 0.001) Between • Significant improvements in balance confidence (*p* < 0.001)
Lu et al., 2016	China	Pilot RCT	*n* = 45 (22 exercise, 23 control); 69.7 years [range NR]; 13M/32F	MCI (*mean* MMSE: 26.83/30; SD: 1.82)	**Momentum-based dumbbell training** (three times per week, 60 min per session, for 3 months = 36 h)	**Community center-based** (Group; in-person; intervention delivered by physical education students; exercises were individually tailored, progressive difficulty, frequent breaks)	Strength exercises	No intervention	Primary • Cognition Secondary • Functional mobility; forward stability; balance confidence	Balance confidence	ABC scale (/100)	• **Pre-:** 94.5 • **Post-:** 93.55	Within • NS (*p*-value NR)Between • NS (*p* = 0.561)
Nyman et al., 2019	United Kingdom	RCT	*n* = 85 (42 exercise, 43 control); 78.1 years [range 59–97.4]; 51M/34F	Dementia (*mean* M-ACE: 15.65/30; SD: 4.6)	**Tai Chi** (once per week, 45 min per session, for 5 months = 15 h)	**Community center-based** (Group and individual; in-person and remote; caregiver involvement + instructor-led sessions; leveraging procedural memory, repetition, positive reinforcement)	Balance, strength, and flexibility exercises	No intervention	Primary • Functional mobility Secondary • Balance; fear of falls; QoL; falls	Fear of falling	iconFES (/120)	• **Pre-:** 16.6 • **Post-:** 17.3	Within • NR Between • NS (*p* = 0.30)
Ofosu et al., 2023	United Kingdom	Feasibility and evaluation study	*n* = 33; 84.9% were 75+ years [range 60–85+ years]; 5M/28F	Dementia (NR)	**Digital music and movement intervention** (frequency NR, session duration NR, for 3 months = total dose NR)	**LTC-based** (Group and individual; in-person and remote due to COVID-19; intervention delivered by care home staff; played music before exercise to improve mood)	Dance (specific exercise types NR)	–	Fear of falling; QoL; psychosocial well-being; sleep; appetite; frailty	Fear of falling	Short FES-I (/28)	• **Pre-:** 8.17 • **Post-:** 8.72	Within • NS (*p* = 0.752) Between • NA
Okuyan and Deveci, 2021	Turkey	RCT	*n* = 42 (20 exercise, 22 control); 74.2 years [range NR]; 27M/15F	MCI (NR)	**Tai Chi** (twice per week, 30–45 min per session, for 3 months = 12–18 h)	**LTC-based** (Group)	Balance, strength, and flexibility exercises	No intervention	Primary • Fear of falling Secondary • Gait; risk of falling; cognition	Fear of falling	TSK (/68)	• **Pre-:** 42.34 • **Post-:** 48.21	Within • Significant change in fear of movement (*p* < 0.001) Between • Significant differences in fear of movement (*p* < 0.001)
Padala et al., 2017	United States of America	Pilot RCT	*n* = 30 (15 exercise, 15 control); 73.0 years [range NR]; 19M/11F	Dementia (*mean* MMSE: 22.9/30; SD: 2.2)	**Wii-Fit video game-led program** (five times per week, 30 min per session, for 2 months = 20 h)	**Home-based** (Individual; supervised by caregivers; progressive increase in difficulty, individually tailored)	Strength, flexibility, aerobic, and balance exercises	Balance exercises	Primary • Balance Secondary • Balance confidence; falls efficacy; QoL	Balance confidence and fall efficacy	ABC scale (/100) • FES (/100)	**ABC** • **Pre-:** 83.2 • **Post-:** 87.9 **FES** • **Pre-:** 16.7 • **Post-:** 13.0	Within • NS (*p*-value NR)Between • Significant improvements in balance confidence (**ABC:** *p* < 0.001) and fall efficacy (**FES:** *p* = 0.002)
Roh and Lee, 2019	Korea	Non-randomized pre- and post-test study	*n* = 18 (9 exercise, 9 control); 76.6 years [range NR]; M/F distribution NR	MCI (*mean* MoCA: 21.06/30; SD: 4.12)	**Gaze stability exercises** (twice per week, 60 min per session, for 1 month = 8 h)	NR	Balance exercises	Gaze stability exercises, but no MCI	Cognition; mobility and balance; balance confidence; subjective health status	Balance confidence	ABC scale (/100)	• **Pre-:** 66.74 • **Post-:** 83.82	Within • Significant improvements in balance confidence (*p* < 0.01) Between • Significant improvements in balance confidence (*p* < 0.05)
Schwenk et al., 2016	United States of America	Pilot RCT	*n* = 22 (12 exercise, 10 control); 78.2 years [range NR]; 10M/12F	MCI (*mean* MoCA: 22.85/30; SD: 3.05)	**Sensor-based balance training with motion feedback** (twice per week, 45 min per session, for 4 weeks = 6 h)	**Outpatient-clinic based** (Individual; delivery NR; accessible graphical interface)	Balance exercises	No intervention	Balance; gait; fear of falling; cognition	Fear of falling	Short FES-I (/28)	• **Pre-:** 9.0 • **Post-:** 8.2	Within • NR Between • Significant reductions in fear of falling (*p* = 0.02)
Taylor et al., 2017	Australia	Quasi-experimental pre- and post-test study	*n* = 42; 83 years [range NR]; 20M/22F	Dementia (*mean* MMSE: 21.2/30; SD: 4.1)	**Carer-enhanced, progressive, and individually tailored exercise program** (6 months; frequency per week and session duration NR = total dose NR)	**Home-based** (Individual; remote; frequent PT visits with progressive, individual tailoring + caregiver involvement)	Strength and balance exercises	–	Primary • Balance; mood Secondary • Fear of falling; physical activity; QoL; hand • reaction time; leg strength; fall risk	Fear of falling	iconFES (/120)	• **Pre-:** 21 • **Post-:** 17	Within • Significant reductions in concerns about falling (*p* = 0.04) Between • NA
Taylor et al., 2021	Australia	RCT	*n* = 309 (153 exercise, 156 control); 82.0 years [range NR]; 158M/151F	Dementia (*mean* M-ACE: 14.4/30; SD: NR)	**Home-based exercise program** **+** **home hazard reduction** (two times per week, 40–60 min per session, for 12 months = 69.68–104 h)	**Home-based** (Individual; remote; caregiver involvement + frequent PT and OT visits + intervention led by PTs; individually tailored, progressive increase in difficulty)	Balance and strength exercises	No intervention	Primary • Rate of falls Secondary • Fall status; physical function; fear of falling; QoL	Fear of falling	iconFES (/120)	• **Pre-:** 18 • **Post-:** 20	Within • NR Between • NS (p = 0.5)
Ullrich et al., 2022	Germany	RCT	*n* = 118 (63 exercise, 55 control); 82.3 years [range NR]; 28M/90F	Dementia (*mean* MMSE: 23.3/30; SD: 2.4)	**Home-based physical training and activity promotion program** (3 months; frequency per week and session length NR = total dose NR)	**Home-based** (Individual; remote; introduced to exercise by academic sports scientists; “dementia-friendly” autonomous exercises supported by tailored motivational strategies and graphical instructions)	Strength and balance exercises	Motor placebo activity	Primary • Physical capacity; physical activity Secondary • Strength; gait; balance; mobility; QoL; fear of falling; depression	Fear of falling	Short FES-I (/28)	• **Pre-:** 12.24 • **Post-:** 10.48	Within • NR Between • Significant difference in concerns about falling (*p* = 0.008)
Uysal et al., 2023	Turkey	Four-arm RCT	*n* = 48 (12 AG, 12 DG, 12 ADG, 12 CG); 73.725 years [range NR]; 40M/8F	MCI (*mean* MMSE: 21.03/30; SD: 1.06)	• **AG** (3 days per week; 30 min per session; for 3 months = 18 h) • **DG** (3 days per week; 30 min per session; for 3 months = 18 h) • **ADG** (3 days per week; 30 min per session; for 3 months = 18 h)	**Outpatient clinic-based** (Individual; in-person; interventions delivered by a trained therapist; exercises individually tailored in intensity and gradually progressed)	Aerobic, strength, balance, and cognitive exercises	Lower limb strengthening exercises (no aerobic or dual-task training)	Primary • Cognition Secondary • Lower limb strength; balance; mobility; balance confidence; functional exercise capacity; physical performance; mood; QoL	Balance confidence	ABC (/100)	**AG** • **Pre-:** 71.25 • **Post-:** 75.26 **DG** • **Pre-:** 70.52 • **Post-:** 75.94 **ADG** • **Pre-:** 71.98 • **Post-:** 77.66	Within • **AG:** Significant increase in balance confidence (*p* < 0.0001) • **DG:** Significant increase in balance confidence (*p* < 0.0001) • **ADG:** Significant increase in balance confidence (*p* < 0.002) Between • NR
Varriano et al., 2020	Canada	Pilot RCT	*n* = 12 (8 exercise, 4 control); 74.3 years [range NR]; 8M/4F	Dementia and MCI (*mean* MoCA: 23.0/30; SD: 1.5)	**Vestibular exercises** (3 times daily, 3–10 min per session, for 3 months = 3.9–6 h)	**Home-based** (Individual; remote; progressive exercises and frequent check-in calls)	Balance exercises	No intervention	Balance; balance confidence; gait; QoL; depression	Balance confidence	ABC scale (/100)	• **Pre-:** 53.4 • **Post-:** 85.3	Within • NR Between • NR
Wesson et al., 2013	Australia	Pilot RCT	*n* = 22 (11 exercise, 11 control); 79.8 years [range NR]; 13M/9F	Dementia (*mean* MMSE: 23.5/30; SD: 3.7)	**Exercise** **+** **home hazard reduction** (three times per week, session length NR, for 3 months = total dose NR)	**Home-based** (Individual; remote; program tailored to participant's individual cognitive levels + caregiver involvement, progressive increase in difficulty, check-in calls)	Strength and balance exercises	No intervention	Primary • Physical function Secondary • Physical activity; fear of falling; falls	Fear of falling	• iconFES (/120) • Short FES-I (/28)	**iconFES** • **Pre-:** 51.6 • **Post-:** 47.3 **Short FES-I** • **Pre-:** 10.5 • **Post-:** 8.2	Within • NR Between • NS (**iconFES:** *p* = 0.56; **Short FES-I:** *p* = 0.71)
Zhang et al., 2021	Australia	Quasi-experimental pre- and post- test study	*n* = 15; 87.5 years [range 76–96]; 8M/7F	Dementia (*mean* RUDAS: 24.2/30; SD: 2.0)	**Safe mobilization program** (1–4 sessions per week, 30 min per session, for 0.75 months = 1.5–6 h)	**Home-based** (Individual; in-person; intervention provided by an OT; graded progression and repeated verbal cues)	Strength and balance exercises	–	Functional mobility; falls; falls risk; fear of falling	Fear of falling	FES-I (/64)	• **Pre-:** 25 • **Post-:** 21.55	Within • Sample size not large enough to test for significance Between • NA

ABC, activities-specific balance confidence; ADL, activities of daily living; ADP, adult day program; ADG, aerobic training, dual-task training, and lower limb strengthening group; AG, aerobic training and lower limb strengthening group; CMMSE, Cantonese Mini-Mental State Examination; CCT, computerized cognitive training; DG, dual-task training and lower limb strengthening group; FES, Falls Efficacy Scale; FES-I, Falls Efficacy Scale (International); HDS-R, Hasegawa's Dementia Scale (Revised); iconFES, Iconographical Falls Efficacy Scale; LTC, long-term care; MCI, mild cognitive impairment; M-ACE, mini-Addenbrooke's cognitive examination; MDRS, Mattis Dementia Rating Scale; MMSE, mini-mental state examination; MMSE-K, mini-mental state examination (Korean); MoCA, montreal cognitive assessment; NA, not applicable; NR, not reported; NS, not significant; OT, occupational therapist; PT, physical therapist; QoL, quality of life; RCT, randomized controlled trial; RUDAS, Rowland Universal Dementia Assessment Scale; Short FES, Short Falls Efficacy Scale; Short FES-I, Short Falls Efficacy Scale (International); SD, standard deviation; TSK, Tampa Scale of Kinesiophobia.

## 3 Results

### 3.1 Descriptive summary

A total of 27 studies were identified that used exercise interventions to help address concerns about falling in people with MCI and dementia (see Table 3). The year of publication spanned 2006 (Kato et al., 2006) to 2023 (Harwood et al., 2023; Hwang et al., 2023; Kiyoshi-Teo et al., 2023; Lee et al., 2023; Ofosu et al., 2023; Uysal et al., 2023), with 1,882 study participants reported (range: 12–365 participants; mean age: 72.9 years; 40.6% female). Only three studies (Harwood et al., 2023; Nyman et al., 2019; Zhang et al., 2021) reported the age range of their participants, so overall age range was not reported. The research occurred in a total of 13 locations (Table 3), with the most common location being Australia (*n* = 6) (Lee et al., 2023; Zhang et al., 2021; Callisaya et al., 2021; Taylor et al., 2017, 2021; Wesson et al., 2013), followed by the United States (*n* = 4) (Li et al., 2021; Kiyoshi-Teo et al., 2023; Padala et al., 2017; Schwenk et al., 2016), and the United Kingdom (*n* = 3) (Harwood et al., 2023; Ofosu et al., 2023; Nyman et al., 2019). Only one study was conducted in Canada (Varriano et al., 2020), where the authors of this review are located. The studies showed substantial heterogeneity in exercise program frequency and duration. For example, the duration of the study interventions ranged from 4 weeks (Roh and Lee, 2019) to 12 months (Taylor et al., 2021), and the frequency of the exercise sessions ranged from once per week (Kiyoshi-Teo et al., 2023; Nyman et al., 2019; Charras et al., 2020; Chen and Pei, 2018) to daily (Hagovska and Olekszyova, 2016). A total of 707.8 h of exercise was performed across the interventions, with a mean total of 30.8 h per study (range: 3.75–156 h).

Of the 27 studies, most (*n* = 16; 59.3%) focused on dementia (Kato et al., 2006; Lee et al., 2023; Ofosu et al., 2023; Nyman et al., 2019; Zhang et al., 2021; Taylor et al., 2017, 2021; Wesson et al., 2013; Padala et al., 2017; Charras et al., 2020; Chen and Pei, 2018; Dechamps et al., 2009; Kim et al., 2017; Lam F. M. H. et al., 2018; Li et al., 2014; Ullrich et al., 2022), eight studies (29.6%) examined people with MCI (Hwang et al., 2023; Uysal et al., 2023; Callisaya et al., 2021; Schwenk et al., 2016; Roh and Lee, 2019; Hagovska and Olekszyova, 2016; Lu et al., 2016; Okuyan and Deveci, 2021), and three studies (Harwood et al., 2023; Kiyoshi-Teo et al., 2023; Varriano et al., 2020) researched both populations. Of the studies focusing on people with dementia, participants were in the early (*n* = 7; 46.7%) (Zhang et al., 2021; Taylor et al., 2017; Wesson et al., 2013; Padala et al., 2017; Dechamps et al., 2009; Li et al., 2014; Ullrich et al., 2022) or moderate stages of the disease (*n* = 8; 53.3%) (Kato et al., 2006; Lee et al., 2023; Nyman et al., 2019; Taylor et al., 2021; Charras et al., 2020; Chen and Pei, 2018; Kim et al., 2017; Lam F. M. H. et al., 2018), as indicated by their scores on baseline cognitive assessments. None of the studies featured participants with more severe dementia. One study looking at people with dementia (Ofosu et al., 2023), plus one study examining people with MCI (Okuyan and Deveci, 2021), did not report participants' baseline cognitive scores. Most studies did not report the range of cognitive scores; therefore, only mean and standard deviation were included in Table 3.

To understand the degree of concerns about falling among the participants, we took the reported scores from the included articles and interpreted them based on the established cut-off scores for each measure (e.g., ABC scale score of 81 or higher indicates high balance confidence). Regarding concerns about falling, only five of 27 studies (18.5%) (Kato et al., 2006; Kiyoshi-Teo et al., 2023; Charras et al., 2020; Chen and Pei, 2018; Kim et al., 2017) included participants with high concerns about falling at baseline. The remaining 22 studies (81.5%) recruited participants with low/no (*n* = 12; 44.4%) (Hwang et al., 2023; Lee et al., 2023; Ofosu et al., 2023; Nyman et al., 2019; Callisaya et al., 2021; Taylor et al., 2017, 2021; Padala et al., 2017; Hagovska and Olekszyova, 2016; Dechamps et al., 2009; Lam F. M. H. et al., 2018; Lu et al., 2016) or moderate (*n* = 10; 37.0%) (Harwood et al., 2023; Uysal et al., 2023; Zhang et al., 2021; Wesson et al., 2013; Schwenk et al., 2016; Varriano et al., 2020; Roh and Lee, 2019; Li et al., 2014; Okuyan and Deveci, 2021; Ullrich et al., 2022) baseline concerns about falling. Twenty studies (74.1%) featured a type of randomized controlled trial (*n* = 19) (Kato et al., 2006; Harwood et al., 2023; Hwang et al., 2023; Uysal et al., 2023; Nyman et al., 2019; Callisaya et al., 2021; Taylor et al., 2021; Wesson et al., 2013; Padala et al., 2017; Schwenk et al., 2016; Varriano et al., 2020; Chen and Pei, 2018; Hagovska and Olekszyova, 2016; Dechamps et al., 2009; Kim et al., 2017; Lam F. M. H. et al., 2018; Lu et al., 2016; Okuyan and Deveci, 2021; Ullrich et al., 2022) or crossover design (*n* = 1) (Charras et al., 2020) with exercise and control or usual care arms. The remaining studies (*n* = 7) consisted of three quasi-experimental (i.e., one arm) pre- and post-test studies (Kiyoshi-Teo et al., 2023; Zhang et al., 2021; Taylor et al., 2017), two non-randomized (i.e., two arm) pre- and post-test studies (Roh and Lee, 2019; Li et al., 2014), and two pilot feasibility studies (Lee et al., 2023; Ofosu et al., 2023).

### 3.2 Detailed findings

#### 3.2.1 Types of exercises

Eighteen of the 27 included studies (66.7%) were multi-modal (Kato et al., 2006; Harwood et al., 2023; Hwang et al., 2023; Lee et al., 2023; Uysal et al., 2023; Nyman et al., 2019; Zhang et al., 2021; Callisaya et al., 2021; Taylor et al., 2017, 2021; Wesson et al., 2013; Padala et al., 2017; Charras et al., 2020; Dechamps et al., 2009; Lam F. M. H. et al., 2018; Li et al., 2014; Okuyan and Deveci, 2021; Ullrich et al., 2022). Balance was the most used type of exercise, included in all but three articles (i.e., *n* = 24; 88.9%; see Table 2) (Kiyoshi-Teo et al., 2023; Ofosu et al., 2023; Lu et al., 2016), two of which did not state the specific types of exercises included in the interventions (Kiyoshi-Teo et al., 2023; Ofosu et al., 2023). Examples of balance exercises included performing specific stances (e.g., one-legged stance, tandem stance) and stepping activities (e.g., side-stepping, step-ups onto a block). Second after balance were strength exercises, used in 18 (66.7%) studies (Kato et al., 2006; Harwood et al., 2023; Hwang et al., 2023; Lee et al., 2023; Uysal et al., 2023; Nyman et al., 2019; Zhang et al., 2021; Callisaya et al., 2021; Taylor et al., 2017, 2021; Wesson et al., 2013; Padala et al., 2017; Dechamps et al., 2009; Lam F. M. H. et al., 2018; Li et al., 2014; Lu et al., 2016; Okuyan and Deveci, 2021; Ullrich et al., 2022), often partnered with balance. In addition to balance and strength, other modalities included flexibility (Kato et al., 2006; Hwang et al., 2023; Nyman et al., 2019; Padala et al., 2017; Dechamps et al., 2009; Li et al., 2014; Okuyan and Deveci, 2021), aerobic fitness (Uysal et al., 2023; Padala et al., 2017), and coordination (Charras et al., 2020). Five studies (18.5%) implemented Tai Chi-based exercise (Hwang et al., 2023; Nyman et al., 2019; Dechamps et al., 2009; Li et al., 2014; Okuyan and Deveci, 2021), simultaneously targeting balance, strength, and flexibility.

#### 3.2.2 Intervention details

Of the 27 included articles, the exercise interventions occurred in a variety of settings serving older adults with MCI and dementia (see Table 3). Personal homes were the most common location for the exercise interventions (*n* = 11; 40.7%) (Harwood et al., 2023; Hwang et al., 2023; Lee et al., 2023; Zhang et al., 2021; Callisaya et al., 2021; Taylor et al., 2017, 2021; Wesson et al., 2013; Padala et al., 2017; Varriano et al., 2020; Ullrich et al., 2022), followed by community-based settings such as private rehabilitation clinics (*n* = 4) (Uysal et al., 2023; Schwenk et al., 2016; Chen and Pei, 2018; Hagovska and Olekszyova, 2016), community centers (*n* = 3) (Nyman et al., 2019; Li et al., 2014; Lu et al., 2016), and adult day programs (*n* = 3) (Charras et al., 2020; Kim et al., 2017; Lam F. M. H. et al., 2018). The remaining studies occurred in institutional settings such as long-term care (*n* = 4) (Kato et al., 2006; Ofosu et al., 2023; Dechamps et al., 2009; Okuyan and Deveci, 2021) and one assisted-living facility (Kiyoshi-Teo et al., 2023). One study did not report information about the intervention setting (Roh and Lee, 2019).

Over half of the studies (14/27; 51.9%) delivered the interventions individually (Harwood et al., 2023; Hwang et al., 2023; Kiyoshi-Teo et al., 2023; Lee et al., 2023; Uysal et al., 2023; Zhang et al., 2021; Callisaya et al., 2021; Taylor et al., 2017, 2021; Wesson et al., 2013; Padala et al., 2017; Schwenk et al., 2016; Varriano et al., 2020; Ullrich et al., 2022). A further third (*n* = 9; 33.3%) were delivered in a group (Kato et al., 2006; Charras et al., 2020; Chen and Pei, 2018; Dechamps et al., 2009; Kim et al., 2017; Lam F. M. H. et al., 2018; Li et al., 2014; Lu et al., 2016; Okuyan and Deveci, 2021). The remaining studies offered both group and individual formats (Ofosu et al., 2023; Nyman et al., 2019), or the format was not reported (Roh and Lee, 2019; Hagovska and Olekszyova, 2016). Individual-based intervention studies tended to be delivered in participants' homes, while group-based interventions tended to take place in long-term care, adult day programs, outpatient rehabilitation clinics, and community centers.

Twelve of the 27 (44.4%) interventions occurred in-person (Kato et al., 2006; Hwang et al., 2023; Kiyoshi-Teo et al., 2023; Uysal et al., 2023; Charras et al., 2020; Chen and Pei, 2018; Hagovska and Olekszyova, 2016; Dechamps et al., 2009; Kim et al., 2017; Lam F. M. H. et al., 2018; Li et al., 2014; Lu et al., 2016), six (22.2%) were delivered remotely (Callisaya et al., 2021; Taylor et al., 2017, 2021; Wesson et al., 2013; Varriano et al., 2020; Ullrich et al., 2022), and four used both in-person and remote approaches. Two studies that used both in-person and remote delivery did so due to challenges associated with the COVID-19 pandemic (Harwood et al., 2023; Ofosu et al., 2023). One-quarter of the studies (*n* = 7) used technology (e.g., touchscreen tablets, music devices, sensor-based exercise platforms, commercial exercise gaming consoles) to deliver the exercise interventions (Ofosu et al., 2023; Callisaya et al., 2021; Padala et al., 2017; Schwenk et al., 2016; Hagovska and Olekszyova, 2016; Lam F. M. H. et al., 2018; Lu et al., 2016). Delivery details of the intervention were not mentioned in four studies (Padala et al., 2017; Schwenk et al., 2016; Roh and Lee, 2019; Okuyan and Deveci, 2021).

#### 3.2.3 Outcome measures

Only one study examined participants' concerns about falling as the primary or first-listed outcome (Okuyan and Deveci, 2021). In the remaining 26 studies, concerns about falling were a secondary outcome (see Table 3). Of the 27 articles, all (100%) examined concerns about falling using self-report, quantitative outcome measures. Six different scales were used in total across the 27 studies, including the ABC scale, which was used the most in just over one-third of studies (*n* = 10; 37%) (Hwang et al., 2023; Uysal et al., 2023; Callisaya et al., 2021; Padala et al., 2017; Varriano et al., 2020; Roh and Lee, 2019; Charras et al., 2020; Lam F. M. H. et al., 2018; Li et al., 2014; Lu et al., 2016), followed by the Short FES-I in six studies (22.2%) (Harwood et al., 2023; Kiyoshi-Teo et al., 2023; Ofosu et al., 2023; Wesson et al., 2013; Schwenk et al., 2016; Ullrich et al., 2022). Other measures used to examine concerns about falling in this population included the FES-I (*n* = 4) (Zhang et al., 2021; Chen and Pei, 2018; Hagovska and Olekszyova, 2016; Kim et al., 2017), iconFES (*n* = 4) (Lee et al., 2023; Nyman et al., 2019; Taylor et al., 2017, 2021), original FES (*n* = 3) (Kato et al., 2006; Padala et al., 2017; Dechamps et al., 2009), and the TSK (*n* = 1) (Okuyan and Deveci, 2021).

The most measured construct related to concerns about falling was fear of falling, identified in half of the studies (*n* = 14; 51.9%) (Harwood et al., 2023; Kiyoshi-Teo et al., 2023; Lee et al., 2023; Ofosu et al., 2023; Nyman et al., 2019; Zhang et al., 2021; Taylor et al., 2017, 2021; Wesson et al., 2013; Schwenk et al., 2016; Chen and Pei, 2018; Hagovska and Olekszyova, 2016; Kim et al., 2017; Ullrich et al., 2022). This construct was measured using several different scales. Next was balance confidence, measured in 10 studies (37.0%) (Hwang et al., 2023; Uysal et al., 2023; Callisaya et al., 2021; Padala et al., 2017; Varriano et al., 2020; Roh and Lee, 2019; Charras et al., 2020; Lam F. M. H. et al., 2018; Li et al., 2014; Lu et al., 2016), continuously assessed by the ABC scale (Powell and Myers, 1995). Fall efficacy was measured in three articles (11.1%) (Kato et al., 2006; Padala et al., 2017; Dechamps et al., 2009); one of these studies also measured balance confidence (Padala et al., 2017). Finally, the one study looking at concerns about falling as the primary outcome (Okuyan and Deveci, 2021) measured fear of movement using the TSK. No studies examined movement confidence as a construct.

#### 3.2.4 Participant accommodations

Several of the studies reported making accommodations for the cognitive needs of the participants with MCI or dementia (see Table 3). The primary accommodation was using “support persons.” In 20 studies (74.1%), interventions were supported by clinicians (e.g., OT, PT) and other trained facilitators (e.g., kinesiology students, nurse practitioners, exercise physiologists) (Kato et al., 2006; Harwood et al., 2023; Hwang et al., 2023; Kiyoshi-Teo et al., 2023; Ofosu et al., 2023; Uysal et al., 2023; Nyman et al., 2019; Zhang et al., 2021; Callisaya et al., 2021; Taylor et al., 2017, 2021; Charras et al., 2020; Chen and Pei, 2018; Hagovska and Olekszyova, 2016; Dechamps et al., 2009; Kim et al., 2017; Lam F. M. H. et al., 2018; Li et al., 2014; Lu et al., 2016; Ullrich et al., 2022) to prescribe, deliver, progress, and/or supervise/monitor the exercise interventions. There was also some degree of family/friend caregiver involvement (*n* = 6; 22.2%) (Lee et al., 2023; Nyman et al., 2019; Taylor et al., 2017, 2021; Wesson et al., 2013; Padala et al., 2017) for additional support during the exercise interventions and related data collection, particularly for home-based programs (e.g., monitoring for safety or motivational purposes).

Another accommodation for participants with MCI and dementia was individual tailoring (*n* = 13; 48.0%) by establishing initial exercises, sets, repetitions, and durations to suit participant abilities (Harwood et al., 2023; Hwang et al., 2023; Kiyoshi-Teo et al., 2023; Lee et al., 2023; Uysal et al., 2023; Callisaya et al., 2021; Taylor et al., 2017, 2021; Wesson et al., 2013; Padala et al., 2017; Hagovska and Olekszyova, 2016; Lu et al., 2016; Ullrich et al., 2022). In addition to, or as part of individual tailoring, progressive increases in difficulty (*n* = 11; 40.7%) (Lee et al., 2023; Uysal et al., 2023; Zhang et al., 2021; Callisaya et al., 2021; Taylor et al., 2017, 2021; Wesson et al., 2013; Padala et al., 2017; Varriano et al., 2020; Hagovska and Olekszyova, 2016; Lu et al., 2016) of exercise components (e.g., narrowing stance, adding weights) were used as participants became increasingly able to perform the exercises initially prescribed. Additional accommodations for participants with MCI and dementia included “cognitive-friendly” instruction techniques (*n* = 8; 29.6%), such as mirroring, repetition, procedural memory-based pedagogies, and practice sessions (Ofosu et al., 2023; Nyman et al., 2019; Schwenk et al., 2016; Charras et al., 2020; Lam F. M. H. et al., 2018; Li et al., 2014; Lu et al., 2016; Ullrich et al., 2022). A few studies also offered check-in calls (*n* = 3) (Zhang et al., 2021; Callisaya et al., 2021; Varriano et al., 2020) or home visits (*n* = 2) (Taylor et al., 2017, 2021).

#### 3.2.5 Components of effective interventions

The 27 studies explored 13 primary outcomes. Nineteen (70.4%) studies reported statistically significant (Kato et al., 2006; Hwang et al., 2023; Kiyoshi-Teo et al., 2023; Lee et al., 2023; Uysal et al., 2023; Taylor et al., 2017; Padala et al., 2017; Schwenk et al., 2016; Roh and Lee, 2019; Charras et al., 2020; Chen and Pei, 2018; Hagovska and Olekszyova, 2016; Dechamps et al., 2009; Kim et al., 2017; Lam F. M. H. et al., 2018; Li et al., 2014; Lu et al., 2016; Okuyan and Deveci, 2021; Ullrich et al., 2022) changes in their primary or first-listed outcome. Primary or first-listed outcomes were grouped into three categories: cognitive-focused outcomes, physical-focused outcomes, and additional outcomes (see Table 4).

**Table 4:** Summarized primary outcome results of the interventions.

**Citation**	**Primary/first-listed outcome**	**Outcome measure**	**Key findings**
**Cognition-focused studies**			
Chen and Pei, 2018	Attentional control	TMT-A	Exercise participants' TMT-A scores significantly improved compared to controls
Hagovska and Olekszyova, 2016	Cognition	MMSE	Improvements were observed in MMSE in favor of exercise
Hwang et al., 2023	Cognition	- TDRS - TICS-M	Exercise participants' cognition improved compared to controls
Kim et al., 2017	Cognition	- MMSE-K - GDS	Cognition improved in both arms
Li et al., 2014	Cognition	MMSE	Exercise participants showed significant MMSE improvement compared to controls
Lu et al., 2016	Cognition	- ADAS-Cog - TMT-B - DST	Exercise participants significantly improved on ADAS-Cog compared to controls
Roh and Lee, 2019	Cognition	MoCA	Cognition improved in both arms
Uysal et al., 2023	Cognition	MMSE	All exercise arms improved in cognition, while controls did not
**Physical-focused studies**			
Callisaya et al., 2021	Gait speed	Accelerations and decelerations via an electric walkway	No significant differences between arms for gait speed
Charras et al., 2020	Gait and balance	- GUG test - SWWT test - One-leg balance test	Improvements were observed for one-leg balance, GUG, and SWWT tests
Kato et al., 2006	Mobility	FIM	Exercise participants maintained their FIM scores, while controls declined
Lam F. M. H. et al., 2018	Functional mobility	TUG test	Significant main effects of time were identified for the TUG
Nyman et al., 2019	Functional mobility	TUG test	No significant differences on the TUG between arms
Padala et al., 2017	Balance	BBS	Significant differences in BBS scores, in favor of exercise
Schwenk et al., 2016	Balance	Changes in postural sway measured via wearable sensors	Sway was significantly reduced in the exercise arm compared to controls
Taylor et al., 2017	Balance	Changes in postural sway measured via sway meters	Participants significantly improved on the tests of sway
Taylor et al., 2021	Rate of falls	Monthly calendars	No significant reduction in rate of falls in either arm
Ullrich et al., 2022	Physical capacity	SPPB	Exercise participants demonstrated greater outcomes on the SPPB than controls
Varriano et al., 2020	Balance	DGI	Sample size too small to perform statistical analysis
Wesson et al., 2013	Physical function	PPA	No significant difference between arms
Zhang et al., 2021	Functional mobility	- GUG test - Tinetti POMA	Not enough power to determine effectiveness of the intervention
**Additional outcome studies**			
Dechamps et al., 2009	Health-related QoL	SF-12	SF-12 scores improved on all trial arms, independent of cognitive status
Harwood et al., 2023	Carer-rated disability in ADLs	DAD	No significant differences between arms for ADLs
Kiyoshi-Teo et al., 2023	Feasibility	- Study consent rate - Retention	The feasibility criteria for retention were met
Lee et al., 2023	Safety and adherence	Exercise diaries	No adverse/serious events or falls occurred
Ofosu et al., 2023	Feasibility	Adherence and safety (attendance tracking)	Low adherence to the intervention (57%)
Okuyan and Deveci, 2021	Fear of movement	TSK	Significant differences in TSK scores in favor of exercise

ADAS-Cog, Alzheimer's Disease Assessment Scale (Cognitive Subscale); ADL, activities of daily living; BBS, Berg Balance Scale; DAD, disability assessment for dementia; DGI, Dynamic Gait Index; DST, digit span test; FIM, functional independence measure; GDS, Global Deterioration Scale; GUG, get up and go; MDRS, Mattis Dementia Rating Scale; MMSE, mini-mental state examination; MMSE-K, Mini-Mental State Examination (Korean); MoCA, montreal cognitive assessment; PPA, physiological profile assessment; QoL, quality of life; SF-12, 12-item short form health survey; SPPB, short physical performance battery; SWWT, stop walking when talking test; TICS-M, modified telephone interview of cognitive status; Tinetti POMA, tinetti performance-oriented mobility assessment; TMT-A, trail-making test (part A); TMT-B, trail-making test (part B); TSK, tampa scale of Kinesiophobia; TUG, timed up and go.

The most common primary outcome among the 27 studies was cognition. Seven studies (25.9%) had a cognitive measure as their primary outcome (Hwang et al., 2023; Uysal et al., 2023; Roh and Lee, 2019; Hagovska and Olekszyova, 2016; Kim et al., 2017; Li et al., 2014; Lu et al., 2016), with one further study (Chen and Pei, 2018) focusing on an aspect of cognition (attentional control). All studies with cognition (or an aspect of it) as their primary outcome showed significant improvements at post-intervention.

Despite the prevalence of balance exercise interventions, only five studies (18.5%) looked at balance as their primary outcome (Taylor et al., 2017; Padala et al., 2017; Schwenk et al., 2016; Varriano et al., 2020; Charras et al., 2020), of which four (Taylor et al., 2017; Padala et al., 2017; Schwenk et al., 2016; Charras et al., 2020) recorded a significant change. Four studies focused on mobility as the primary or first-listed outcome (Kato et al., 2006; Nyman et al., 2019; Zhang et al., 2021; Lam F. M. H. et al., 2018), with only one demonstrating a significant effect at post-intervention (Lam F. M. H. et al., 2018). Of the two studies primarily examining gait (Callisaya et al., 2021; Charras et al., 2020), only one significantly improved (Charras et al., 2020). The one study looking at fall rate (Taylor et al., 2021) was not significant at post-intervention, while the single study investigating physical capacity [defined as “the ability to perform a physical task or action measured by self-report or objective measurement” (Kasper et al., 2017)] was (Ullrich et al., 2022).

Other primary outcomes concerned the feasibility of the exercise intervention (*n* = 2) (Kiyoshi-Teo et al., 2023; Ofosu et al., 2023), one of which was significant (Kiyoshi-Teo et al., 2023), followed by safety and adherence (*n* = 1; criteria met) (Lee et al., 2023), ADLs (*n* = 1; not significant) (Harwood et al., 2023), health-related QoL (*n* = 1; significant at post-intervention) (Dechamps et al., 2009), and fear of movement (*n* = 1; significant at post-intervention) (Okuyan and Deveci, 2021). Two studies (Zhang et al., 2021; Varriano et al., 2020) did not have a large enough sample to perform statistical analyses and could not examine the effectiveness of the exercise interventions on their primary outcome(s).

Of the 19 “successful” interventions (i.e., the intervention significantly impacted the primary outcome), most (*n* = 13; 68.4%) were conducted outside of the home setting (e.g., LTC, ADPs, outpatient clinics, community centers, etc.) (Kato et al., 2006; Kiyoshi-Teo et al., 2023; Uysal et al., 2023; Schwenk et al., 2016; Charras et al., 2020; Chen and Pei, 2018; Hagovska and Olekszyova, 2016; Dechamps et al., 2009; Kim et al., 2017; Lam F. M. H. et al., 2018; Li et al., 2014; Lu et al., 2016; Okuyan and Deveci, 2021). There was a relatively even split between group (*n* = 9/19; 47.4%) (Kato et al., 2006; Charras et al., 2020; Chen and Pei, 2018; Dechamps et al., 2009; Kim et al., 2017; Lam F. M. H. et al., 2018; Li et al., 2014; Lu et al., 2016; Okuyan and Deveci, 2021) and individual (*n* = 8/19; 42.1%) interventions (Hwang et al., 2023; Kiyoshi-Teo et al., 2023; Lee et al., 2023; Uysal et al., 2023; Taylor et al., 2017; Padala et al., 2017; Schwenk et al., 2016; Ullrich et al., 2022), with two studies (Roh and Lee, 2019; Hagovska and Olekszyova, 2016) not reporting this information. Many successful interventions (*n* = 13/19; 68.4%) were conducted in-person (Kato et al., 2006; Hwang et al., 2023; Kiyoshi-Teo et al., 2023; Lee et al., 2023; Uysal et al., 2023; Charras et al., 2020; Chen and Pei, 2018; Hagovska and Olekszyova, 2016; Dechamps et al., 2009; Kim et al., 2017; Lam F. M. H. et al., 2018; Li et al., 2014; Lu et al., 2016) vs. remotely (*n* = 4/19; 21.1%) (Lee et al., 2023; Taylor et al., 2017; Padala et al., 2017; Ullrich et al., 2022). All but two successful studies (Roh and Lee, 2019; Okuyan and Deveci, 2021) (i.e., *n* = 17/19; 89.5%) included at least one form of participant accommodations (e.g., trained instructors, tailored instructions, progressive increase in difficulty, etc.). Of note, in all five studies where participants had a high fear of falling (Kato et al., 2006; Kiyoshi-Teo et al., 2023; Charras et al., 2020; Chen and Pei, 2018; Kim et al., 2017), the exercise interventions significantly impacted their primary or first-listed outcome. These outcomes included mobility (Kato et al., 2006), feasibility (Kiyoshi-Teo et al., 2023), gait and balance (Charras et al., 2020), and cognition (Chen and Pei, 2018; Kim et al., 2017). The intervention dose, including the frequency (i.e., number of sessions per week) and duration (i.e., number of months), did not appear to impact whether the intervention was successful at addressing the primary outcome. For example, of the 19 successful studies, intervention frequency ranged from once weekly (Kiyoshi-Teo et al., 2023; Charras et al., 2020; Chen and Pei, 2018) to daily (Hagovska and Olekszyova, 2016), while the intervention duration ranged from 4 weeks (1 month) (Schwenk et al., 2016; Roh and Lee, 2019) to 24 weeks (6 months) (Hwang et al., 2023; Taylor et al., 2017; Charras et al., 2020; Dechamps et al., 2009). Interestingly, both studies with interventions lasting 1 year (Harwood et al., 2023; Taylor et al., 2021) did not significantly impact their primary outcomes. Additionally, when reviewing level of cognition (i.e., MCI or dementia) in greater detail, it was found that interventions appeared to elicit greater benefit for individuals living with MCI, with only one study (Callisaya et al., 2021) of the eight focusing solely on MCI (Hwang et al., 2023; Uysal et al., 2023; Callisaya et al., 2021; Schwenk et al., 2016; Roh and Lee, 2019; Hagovska and Olekszyova, 2016; Lu et al., 2016; Okuyan and Deveci, 2021) not producing significant impacts (i.e., 7/8 = 87.5%). Finally, but importantly, of the 19 interventions to successfully impact their primary outcome, all but three studies (Kato et al., 2006; Lam F. M. H. et al., 2018; Lu et al., 2016) (*n* = 16/19; 84.2%) also had a significant within- or between-group effect on concerns about falling.

## 4 Discussion

This scoping review mapped exercise interventions to help address concerns about falling in people with MCI and dementia. Of the 27 studies identified, only one (Okuyan and Deveci, 2021) had concerns about falling as the primary outcome. The other 26 included concerns about falling as secondary outcomes. Among the 27 studies, only five included participants who had high concerns about falling, limiting our understanding of the potential of exercise interventions to impact people with MCI and dementia with the most concerns. Therefore, interpreting the potential of the significant studies should consider the degree of concerns about falling (i.e., none, low, medium, or high) among study participants.

Two-thirds of the studies included people living with dementia and one-third with MCI (three included both). Based on reported cognitive scores, the participants were mainly at the mild to moderate stage of dementia. As such, they may have been able to self-report their concerns about falling more accurately, given that insight decreases with the increasing severity of cognitive impairment (McDaniel et al., 1995; Zanetti et al., 1999). The iconFES (30 items and scored out of 120 points), used in only four included studies (Delbaere et al., 2011), includes pictures to assess fear of falling. However, although designed with people with cognitive impairment in mind (Delbaere et al., 2013), the clinical cut-off scores for the iconFES are rather broad, with ≤ 40 indicating low concern about falling, 41–58 indicating moderate concerns about falling, and ≥59 indicating high concerns about falling (Delbaere et al., 2013, 2011). More research is needed to use this tool clinically and, more specifically, to evaluate the effectiveness of exercise interventions in this population.

Eight of the 27 studies had cognition as the primary outcome, all reporting significant improvements from the exercise interventions. Furthermore, four studies reported a significant improvement in balance, supporting the potential of exercise interventions to tackle the two main fall risk factors in dementia (Hsu et al., 2012; Mazoteras Muñoz et al., 2010). Regarding concerns about falling, 16 studies that reported successful interventions also reported a significant within- or between-group effect on concerns about falling (Hwang et al., 2023; Kiyoshi-Teo et al., 2023; Lee et al., 2023; Uysal et al., 2023; Taylor et al., 2017; Padala et al., 2017; Schwenk et al., 2016; Roh and Lee, 2019; Charras et al., 2020; Chen and Pei, 2018; Hagovska and Olekszyova, 2016; Dechamps et al., 2009; Kim et al., 2017; Li et al., 2014; Okuyan and Deveci, 2021; Ullrich et al., 2022). Multimodal interventions (i.e., more than one exercise type) were the most successful.

Multiple studies implemented MCI- and dementia-friendly accommodations. These included support persons, repeated instructions, mirroring, procedural cues, plus elements commonly seen in rehabilitation programs, such as individual tailoring and progressive increases in difficulty. The success of these accommodated interventions (17/19; 89.5%) challenges the negative perceptions of people living with progressive cognitive impairment to benefit from rehabilitation interventions. The findings also coincide with the development of resources for exercise providers to offer sessions to people living with dementia, such as the “Dementia Inclusive Choices for Exercise” (DICE) toolkit (Middleton et al., 2023). The DICE toolkit includes individual tailoring and progressive increases in difficulty, as well as dementia-friendly instruction techniques (e.g., pacing speech, breaking exercises into steps, offering modifications of exercises) to support exercise providers. These findings demonstrate that people with MCI and dementia can participate in exercise interventions, especially when accommodations are put in place.

The review highlights the current lack of focus on concerns about falling as a primary outcome for exercise targeting people with MCI and dementia. There are a few potential reasons why there is a lack of primary focus on exercise to help address concerns about falling in people with MCI and dementia. Several studies recruited people with MCI and dementia who had little to no concerns about falling at baseline, which may have impacted the degree to which participants could improve because of the intervention. For example, participants with MCI in Callisaya et al. (2021) scored a mean of 87.9/100 on the baseline ABC, with higher scores on the ABC indicating higher balance confidence. With scores indicating a high or moderate level of functioning/confidence, researchers may be “missing the true target” regarding who they recruit for exercise interventions. This is important to note given that concerns about falling are associated with fall risk factors among older adults without cognitive impairment, such as poor perceived health and a history of multiple falls (Zijlstra et al., 2007), plus lower muscle strength and worse balance (Deshpande et al., 2009). Future research should explore these associations further with people who have cognitive impairment, as this may help drive more targeted interventions for falls, which people with cognitive impairment are more likely to experience (Meuleners et al., 2016).

This study has some limitations. It is acknowledged that terms encompassing concerns about falling (e.g., balance confidence, fear of falling, etc.) are unique constructs that are defined, measured, and interpreted differently (Adamczewska and Nyman, 2018; Soh et al., 2021). However, this review combined all these terms in the outcome column of the search strategy. This decision could be considered a limitation, creating heterogeneity, or further perpetuating generic mixing of specific terminology. However, combining all terms in one outcome category allowed the review to map the breadth of literature on all constructs related to concerns about falling, which aligns with the objectives of a scoping review (Arksey and O'Malley, 2005). During the review process, the decision was made to exclude any literature not featuring full-text, original, empirical research articles. This excluded gray literature (although none was identified), 11 conference abstracts, one protocol paper, and three review papers. Additionally, there is no “gold-standard” mechanism to measure concerns about falling; this outcome is assessed using self-report measures, as evident by the studies included in this review. With self-report measures assessing concerns about falling and other constructs (e.g., health service use), there is a risk of recall bias or reporting errors when working with people living with cognitive impairment, such as MCI and dementia (Callahan et al., 2015; Frank et al., 2011). To address this, we recommend that future studies use self-report measures suitable for people living with cognitive impairment by offering visual (i.e., picture) cues and prompts, such as the iconFES (Delbaere et al., 2013, 2011). Further, concerns about falling, which are measured through self-report scales, introduce the possibility of social desirability bias, whereby participants modify their responses to be viewed more favorably by others (e.g., researchers) (Delbaere et al., 2010). To mitigate this effect, it is proposed to implement more anonymous, self-administered measurement methods for assessing concerns about falling. Finally, only studies written in English were included in this review, meaning that studies meeting the inclusion criteria but from other languages were excluded.

Despite the limitations identified above, it is anticipated that the conclusions of this review will stimulate future impactful research in this area. For example, there is a clear need for more large-scale, in-depth experiments evaluating exercise interventions involving people living with MCI and dementia, where concerns about falling are the primary outcome. Further, this scoping review confirms the need for a future systematic review on this topic area, where the formal effectiveness of exercise interventions for people living with MCI and dementia on concerns about falling, fall risk factors (e.g., balance), and falls are examined in greater detail. For future studies, it is recommended that researchers use consistent terminology and mechanisms of intervention measurement (e.g., specific scales). For example, in line with the findings of this review regarding the applicability of accommodations for people living with MCI and dementia, it is recommended that researchers adopt the use of the iconFES (Delbaere et al., 2013) with this population given the included visual prompts that generate more reliable responses and reduce the cognitive demands on participants.

## 5 Conclusions

This is the first scoping review to map the literature using exercise interventions to target concerns about falling in people with MCI and dementia. This scoping review highlights that concerns about falling can be improved with exercise interventions in older adults with MCI and dementia. Although concerns about falling were not the primary outcome of most papers, the results also highlight the potential of exercise interventions to target other fall risk factors (e.g., balance) in people with MCI and dementia.

## Data Availability

The original contributions presented in the study are included in the article/supplementary material, further inquiries can be directed to the corresponding author.
